# Exploring the Frequency and Distribution of Ecological Non-monotonicity in Associations among Ecosystem Constituents

**DOI:** 10.1007/s10021-023-00867-9

**Published:** 2023-08-14

**Authors:** Maximilian Hanusch, Xie He, Stefan Janssen, Julian Selke, Wolfgang Trutschnig, Robert R. Junker

**Affiliations:** 1 Department of Environment and Biodiversity, Paris-Lodron-University Salzburg, 5020 Salzburg, Austria; 2 Algorithmic Bioinformatics, Justus-Liebig-University Giessen, 35390 Giessen, Germany; 3 Department for Artificial Intelligence & Human Interfaces, Paris-Lodron-University Salzburg, 5020 Salzburg, Austria; 4 Evolutionary Ecology of Plants, Department of Biology, Philipps-University Marburg, 35043 Marburg, Germany

**Keywords:** bacteria, ecological non-monotonicity, ecosystem coupling, environment, fungi, glacier forefield, nonlinear associations, plants, succession

## Abstract

Complex links between biotic and abiotic constituents are fundamental for the functioning of ecosystems. Although non-monotonic interactions and associations are known to increase the stability, diversity, and productivity of ecosystems, they are frequently ignored by community-level standard statistical approaches. Using the copula-based dependence measure *qad,* capable of quantifying the directed and asymmetric dependence between variables for all forms of (functional) relationships, we determined the proportion of non-monotonic associations between different constituents of an ecosystem (plants, bacteria, fungi, and environmental parameters). Here, we show that up to 59% of all statistically significant associations are non-monotonic. Further, we show that pairwise associations between plants, bacteria, fungi, and environmental parameters are specifically characterized by their strength and degree of monotonicity, for example, microbe–microbe associations are on average stronger than and differ in degree of non-monotonicity from plant–microbe associations. Considering directed and non-monotonic associations, we extended the concept of ecosystem coupling providing more complete insights into the internal order of ecosystems. Our results emphasize the importance of ecological non-monotonicity in characterizing and understanding ecosystem patterns and processes.

**Supplementary Information:**

The online version contains supplementary material available at 10.1007/s10021-023-00867-9.

## Highlights

- Ecological non-monotonicity is often ignored despite its importance in ecosystem stability, diversity, and productivity.
- Up to 59% of all significant associations in a natural ecosystem are non-monotonic.
- Associations between ecosystem constituents exhibit distinct degrees of strength and non-monotonicity.

## Introduction

**Figure B1.1:**
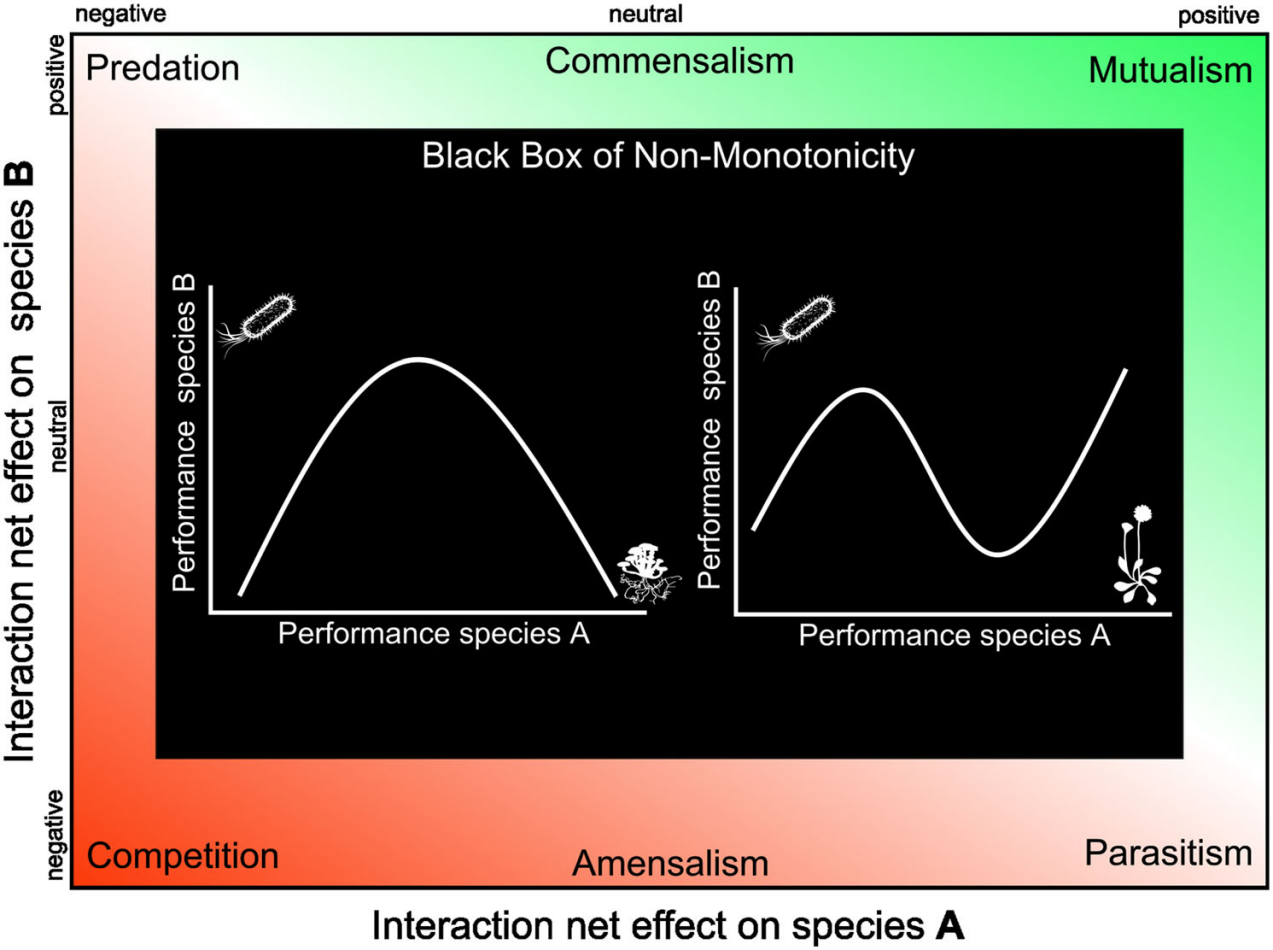
Ecological interaction space assigning species’ interactions into different types ranging from mutualism to competition characterized by net effects on species A and B. Usually, monotonicity is assumed when assigning species interactions into interaction types, where the relationship between species A and B follows a consistent, directional pattern (colored frame). A lack of monotonic dependencies between species was usually interpreted as neutralism. However, the high frequency of non-monotonic associations (as detected in this study) challenges the traditional assumption of strictly monotonic relationships between species and allows first insights into the “black box” of non-monotonicity. Such complex non-monotonic associations between the performance (that is abundance) of two species have been hypothesized in previous studies (for example Maestre and Cortina 2004; Zhang and others 2015; Hirano and Takemoto 2019) but were not considered in the classical interaction types as depicted in the colored frame. Left graph in black box: non-monotonic, dome-shaped association between a taxonomically not resolved fungus on the x-axis and a bacterium of the Acidobacteriaceae family on the y-axis (see Figure 2B). Right graph in black box: oscillating association between the plant species *Scorzoneroides helvetica* on the x-Axis and a Betaproteobacterium on the y-axis (see Figure 2B). These examples emphasize the need to expand the ecological interaction space by considering ecological non-monotonicity.

Natural ecosystems are composed of co-existing organisms that interact with each other and the surrounding environment (Odum 1969). Biotic and abiotic interactions vary in their extent (weak or strong) as well as in their net effect (positive or negative) defining mutualism or antagonism in species interactions. However, interactions may change signs and their net effects may not be either positive or negative under all conditions over time (Figure B1.1 in Box 1). For instance, competition between alpine plant species shifts to facilitation under abiotic stress conditions (Callaway 2002) and outcomes of interactions have been shown to vary with population densities in plant–herbivore and microbe–microbe relationships (Breton and Addicott 1992; Palmer and Foster 2022). Such interactions are, in these cases, not linear or monotonic, but follow other, often unknown non-monotonic and noisy distributions (Box 1). Neither in large nor in small datasets, non-monotonic interactions are captured by standard statistical approaches based on correlation or regression methods (Ghosh 2020a). Accordingly, non-monotonic associations have been largely ignored, despite their vital importance for complex ecosystems (De’Ath and Fabricius 2000; Morales-Castilla and others 2015; Zhang and others 2015, 2020). In fact, non-monotonic interactions increase the stability of ecological networks (Yan and Zhang 2014) as well as the diversity and productivity of ecosystems (Yan and Zhang 2018b). Thus, ignoring non-monotonic dependences may lead to an “oversimplification of ecological complexity” (Zhang and others 2015), which has been widely criticized (for example Coenen and Weitz 2018). As a consequence, additional statistical tools that are able to detect and quantify monotonic as well as non-monotonic dependence have been demanded (Morales-Castilla and others 2015; Hirano and Takemoto 2019). One approach that provides a complete description of associations between two variables is the concept of copulas, which has been successfully applied in other disciplines but only rarely in ecology (Anderson and others 2019; Ghosh and others 2020b; Griessenberger and others 2022; Liebscher and others 2022). A bivariate copula captures the dependence of two random variables *X* and *Y* in a scale-free way, that is, scale changes of the random variables do not affect the copula. In a nutshell, copulas can be viewed as distribution functions of uniformly [0,1]-distributed random variables. According to Sklar’s theorem, bivariate distribution functions can be decomposed into the dependence structure (that is the copula) and the marginal distributions of the random variables (Anderson and others 2019; Ghosh and others 2020a). Recently, the copula-based dependence measure *qad* has been introduced (available as R-package *qad*, Griessenberger and others 2021), which quantifies the directed and asymmetric dependence of two variables including monotonic and non-monotonic associations (see Box 2). In this study, we additionally introduce the index of monotonicity *m* that provides information on the degree of monotonicity of a given association (that is whether the association between *X* and* Y* is rather positively monotonic, non-monotonic, or negatively monotonic. For details, see Box 2) and use it in combination with the directed dependence to characterize associations between ecosystem constituents. Thus, *qad* allows measuring the strength (*q*) and the degree of monotonicity (*m*) independent of each other and can provide a complete characterization of pairwise associations within an ecosystem.

Recently, untargeted, data-driven approaches, such as environment-wide or microbiome-wide association studies (Gilbert and others 2016; Zheng and others 2020) are increasingly applied to explore relationships between numerous ecological variables and species’ abundances, for instance, to screen for beneficial plant-associated microbial strains (Buchholz and others 2021; Zhang and others 2021; Zeng and others 2022). The validity of such approaches relies to a high extent on the choice of the statistical method quantifying the strength of associations (Xia 2020). The inclusion of direction and asymmetry in such data-driven research approaches will provide additional relevant information on bivariate associations (Coenen and Weitz 2018; Ochoa-Hueso and others 2021). Associations between species may be directed (and thus asymmetric) in the sense that a given taxon is facilitating or inhibiting the growth of another taxon without being affected by the other one. Likewise, the net effect of interactions may change, either under the influence of a shared extrinsic factor or through intrinsic effects (Box 1), that is, non-monotonic, context dependent interactions that are notoriously hard to capture by standard statistical approaches (Chamberlain and others 2014; Zhang and others 2020). In this regard, *qad* may serve as a valuable tool for exploring previously unrecognized associations in ecological datasets and for generating more concise hypotheses about underlying latent processes. For instance, microbial communities are, among others, characterized by complex nonlinear associations between interacting species and traditional correlation methods fail to detect these associations, hampering the construction of accurate networks and prediction of biotic dependencies within communities (Hirano and Takemoto 2019; Palmer and Foster 2022).

Acknowledging and quantifying non-monotonicity may advance the recent concept of “ecosystem coupling,” which is regarded as a proxy for the spatial and temporal self-organization of a system and is measured as the systemic strength of associations among biotic and abiotic components of an ecosystem (Ochoa-Hueso and others 2021). Studies investigating the coupling of natural ecosystems have so far used symmetric dependence measures such as Spearman’s rank correlation *rho* to calculate ecosystem coupling (for example Risch and others 2018; Qin and others 2022; Resch and others 2022). Although these approaches greatly deepened our understanding on the links between ecosystem coupling and functioning, they provide a rather static and simplified view on the complexity and underlying structure of natural ecosystems as they are blind to non-monotonic associations (Ochoa-Hueso and others 2021). Especially when dealing with temporal and spatial data or when analyzing ecosystem recovery after severe disruptive events, such as the initiation of primary succession, nonlinear feedbacks and threshold dynamics are important drivers of ecosystem complexity that will lead to numerous non-monotonic associations between ecosystem constituents (Ochoa-Hueso 2016; Hanusch and others 2022). Acknowledging non-monotonic associations provides a more complete picture of the interconnectedness of abiotic and biotic constituents in natural ecosystems. Additionally, with *qad* the concept of ecosystem coupling can be extended toward including the direction of dependence in the underlying associations. Incorporating directional dependence allows to calculate a directed coupling which will permit more refined insights into the relationships among different compartments of the ecosystem and help to generate more precise hypotheses about the underlying processes that define the functioning of natural ecosystems.

In this study, we apply the dependence measure *qad* to capture community-wide, pairwise associations among plants, bacteria, fungi and characteristics of the surrounding environment along a successional gradient in the Austrian Alps to test the following hypotheses and expectations:

1. We conjecture that non-monotonic associations between pairs of biotic and abiotic ecosystem constituents are frequent (compare to Box 1) highlighting the complexity of associations in ecosystems.
2. Given the specific nature of and the different mechanisms underlying pairwise associations between ecosystem constituents (that is association types such as plant–microbe, microbe–microbe, or plant–environment), we hypothesize that these association types are characterized by specific strengths and degrees of monotonicity, leading to association type-specific profiles.
3. Accordingly, we expect variable degrees of directed coupling between ecosystem constituents indicating stronger coupling between some constituents than between others. The detection of directed coupling allows more complete insights into the internal order of ecosystems.

Box 1: Non-monotonic interactions Non-monotonic relationships between species (that is associations that change signs between positive and negative) can be observed in populations, communities, and ecosystems and are often closely related to spatial, temporal or demographic processes that change the biotic or abiotic context in which organisms occur and interact (Ewald 1987; Thompson 1988; Johnson and others 1997). Numerous case studies have reported non-monotonic associations between specific species-pairs beyond classical interaction types (Figure B1.1; Zhang and others 2015), yet the overall extent and distribution of non-monotonic associations in natural systems have been poorly documented (Chamberlain and others 2014). Theoretical work and simulations, however, suggest that ecological non-monotonicity is a key ecosystem component that positively contributes to ecosystem diversity (Gross 2008), stability (Yan and Zhang 2018a), and productivity (Yan and Zhang 2018b). The mechanisms behind non-monotonic patterns in ecological relationships are not fully understood and probably case-specific (Neuhauser and Fargione 2004; Karst and others 2008; Maron and others 2014). However, multiple explanations for this phenomenon have been proposed that can broadly be categorized into the effects of endogenous and exogenous factors (Zhang and others 2015).

Endogenous effects relate to changes in population density (Zhang and others 2020), or variation in trait frequency (Moran and others 2022) of interacting species that can lead to non-monotonic interactions under constant environmental conditions. Specific examples of such endogenously driven changes in individual species interactions are frequently reported by case studies (Breton and Addicott 1992; Dickie and others 2005; Brown and others 2012; Chamberlain and others 2014). Exogenous effects on the other hand, relate to shifts in species–species or species–environment associations caused by changing environmental conditions or under the presence of a third species. For instance, species-specific environmental requirements will lead to shifts from increasing to decreasing occurrence frequencies along strong environmental gradients (Blanchet and others 2020). Further, interactions between species can change sign with increasing environmental stress, as shown in plant–plant (Callaway and others 2002; Maestre and Cortina 2004; Holzapfel and others 2006), plant–microbe (Neuhauser and Fargione 2004; Thrall and others 2007; Hoeksema and others 2010) or microbe–microbe (Palmer and Foster 2022) interactions. Changes in interaction signs are often explained with biological market models: The provision of a beneficial good for a mutualistic partner is always associated with a cost for the provider and is not truly altruistic (“mutual exploitation for mutual gain”; Noë and others 1991; Bronstein 1994; Schwartz and Hoeksema 1998). If the cost of production exceeds the benefit for at least one partner (for example when environmental stress becomes either extremely high or low), then the other partner will begin to preclude or potentially exploit the interspecific relationship (Tielbörger and Kadmon 2000). Another exogenous factor, the presence of a third interacting partner, can lead to changes in species–species relationships (Chamberlain and others 2014). For instance, it is possible for one mutualistic partner to affect the resistance of the other mutualistic partner to predation by sending visual or chemical cues to a predator (Buckley and Ebersole 1994) or predators may have an effect on the interaction outcome of two partners by reducing population densities of one partner after a predation event. In such cases, a change in the interaction outcome does not necessarily imply a change in the mechanism of the interaction but a change in the fitness of interacting partners. However, co-occurring species do not necessarily interact with another. Non-monotonic associations between occurrence frequencies may also arise when species with slightly different ecological requirements co-occur along environmental gradients. Such specific overlaps in occurrence probabilities may also be reflected in non-monotonic associations between independent species (Blanchet and others 2020). Such non-monotonic patterns in species associations could so far not be detected on a large scale with traditional statistical methods. Using *qad* (see Box 2), such relationships can now be detected and quantified in large datasets allowing us to further investigate case-specific mechanisms that govern non-monotonic relationships and the importance of ecological non-monotonicity in general.

## Methods

**Study Design** The study was conducted in the long-term ecological research platform Ödenwinkel which was established in 2019 in the Hohe Tauern National Park, Austria (Dynamic Ecological Information Management System—site and dataset registry: https://deims.org/activity/fefd07db-2f16-46eb-8883-f10fbc9d13a3, last access: November 2022; Junker and others 2020). A total of *n* = 135 permanent plots were established within the glacier forefield of the Ödenwinkelkees, which was covered by ice at the latest glacial maximum in the Little Ice Age around 1850. The plots represent a successional gradient spanning over 1.7 km in length at an altitude ranging from 2070 to 2170 m a.s.l. with a minimum distance of 5 m between plots. Plots were defined as squares with an area of 1 m^2^ and were all oriented in the same cardinal direction. More detailed descriptions of the research platform, exact plot positions, and details on the surrounding environment can be found in Junker and others (2020) and Hanusch and others (2022).

**Biotic and abiotic sampling** Over the vegetation period in 2019, we identified all vascular plant species present on the plots and estimated their cover with a resolution of 0.1%. We sampled soil inhabiting bacteria and fungi from soil cores from an approximate soil depth of 3 cm. Microbial DNA was stabilized and subsequently isolated using Xpedition Fungal/Bacterial DNA MiniPrep (Zymo Research, Freiburg, Germany) following the manufacturer’s instructions. Amplicons of the v3v4 region of the bacterial 16S rRNA gene and the fungal ITS2 region were generated by Eurofins Genomics GmbH (Ebersberg, Germany) prior to sequencing on Illumina MiSeq. Microbiome profiling was performed on the *Qiita* web platform (Gonzalez and others 2018) on which bacterial ASV were obtained using Deblur (Amir and others 2017) and fungal ASV were obtained using DADA2 (Callahan and others 2016). Prior to the statistical analysis of microbial communities, we performed a cumulative sum scaling (CSS) normalization (R-package “metagenomeSeq” v1.28.2, Paulson 2014) on the count data to account for differences in sequencing depth among samples. Soil temperature was measured by installing temperature loggers (MF1921G iButton, Fuchs Elektronik, Weinheim, Germany) 10 cm north of each plot center, at the same depth of 3 cm at which the microbial samples were taken. In 2020, soil samples were taken and soil nutrients (Ca, P, K, Mg, and total N2) as well as soil pH were measured on all plots by AGROLAB Agrar und Umwelt GmbH (Sarstedt, Germany). Detailed information on the sampling strategy of biotic and abiotic parameters can be found in Junker and others (2020) and Hanusch and others (2022).

**Community-wide qad-based association analysis** For the community-wide association analysis, we only considered variables with *n* ≥ 16 unique values along the successional gradient using the R-package *qad* v1.0.3 (Box 2). Additionally, we only considered associations of a pair of variables that co-occurred on at least *n* = 16 plots. This pruning step was necessary 1) in order to avoid a resolution < 4 in the calculation of *qad*, which is defined as square root of the sample size (as recommended by (Junker and others 2021a) and (Griessenberger and others 2022) and 2) in order to only consider meaningful ecological associations: One of the main issues raised in ecological co-occurrence analyses is the erroneous interpretation of spurious co-occurrences (or the lack thereof) that may arise by chance and do not provide meaningful ecological information (Blanchet and others 2020). By pruning our dataset, we avoided the inclusion of spurious missing co-occurrences (that is seemingly competitive exclusion) in our analyses and assured an adequate sampling quantity that allows an ecological interpretation of species associations. We estimated significance levels of the associations with 999 non-parametric permutational steps (as implemented in *qad*, Junker and others 2021a) to address alpha inflation in our analysis (Winkler and others 2016) and consequently removed all non-significant (*p* > 0.05) associations from the resulting dependence matrix.

In total, the final dataset consisted of* n* = 7 abiotic variables, *n* = 30 plant species, *n* = 189 fungal ASVs, and *n* = 1735 bacterial ASVs, from here on referred to as ecosystem constituents (see Supplementary Data 1 for a complete overview of the dataset). We quantified the pairwise asymmetric dependence between all ecosystem constituents by calculating the dependence value *q* for both directions (X,Y) and (Y,X). In contrast to symmetric correlation-based measures, each *qad* calculation results in two directed (that is asymmetric), usually not identical values. For instance, the association between a fungal ASV and a plant species yields the two directed associations of Fungus → Plant and Plant → Fungus, as opposed to the symmetric estimate Plant ↔ Fungus resulting from conventional correlation measures. We calculated all directed dependencies and assigned each association to specific pairs of ecosystem constituents. We refer to these assigned associations as “association types” in the following (for example the directed dependence between a plant and a fungus is assigned the two association types Plants → Fungi and Fungi → Plants).

**Index of monotonicity m** While the dependence estimator *q* informs about the strength of dependence, it does not provide any information on the form of the dependence structure. However, the degree of monotonicity of an association (that is whether an association is positively or negatively monotonic, or non-monotonic) is valuable information for ecologically interpreting given associations. We thus introduce the index of monotonicity *m* that provides an estimate on the degree of monotonicity by capturing the deviation of a given association from ideal monotonicity (a.k.a. co-monotonicity in the positive, and counter-monotonicity in the negative setting) in the same way as *q* quantifies deviation from independence (Trutschnig 2011, for a thorough description of *m* see Box 2). The index of monotonicity *m* is a continuous measure of monotonicity attaining values in the interval [-1,1]. To classify the associations into positively monotonic, negatively monotonic, or non-monotonic, we used Spearman’s rank correlation to assign significant *qad*-based associations either to the group of negative monotonic associations (significant according to *qad*, *m* < 0, significant according to Spearman’s rank correlation, negative rho), positive monotonic associations (significant according to *qad*, *m* > 0, significant according to Spearman’s rank correlation, positive rho), or non-monotonic associations (significant according to *qad*, *m* ~ 0, not significant according to Spearman´s rank correlation).

**Test for proportion of non-monotonic associations** Data on the abundance of taxa as well as abiotic conditions have been sampled along a successional gradient that makes non-monotonic associations more likely to arise because of changing biotic and abiotic conditions over succession as well as because of increasing ecosystem complexity (see Box 1). Thus, we expect more monotonic associations under relatively constant environmental conditions (that is in smaller fractions of the gradient) as well as in less complex and diverse ecosystems (that is in early successional stages). To test whether the proportion of non-monotonic associations increases with (a-)biotic heterogeneity, we divided the successional gradient in plots representing early successional stages (*n* = 68 plots) and those representing late successional stages (*n* = 67 plots). We performed a paired Wilcoxon test to compare the proportion of monotonic and non-monotonic associations among the whole gradient and the early and late successional stages. We further performed a pairwise G-test of independence to test whether the relative proportions of positive and negative monotonic, and non-monotonic associations differ between association types along the total successional gradient.

**Association profiles and their overlap among association types** We characterized each pairwise association by its strength *q* and its index of monotonicity *m* allowing to project each association into a two-dimensional “association space” using *q* and *m* as coordinates. The distribution of associations in the two-dimensional space visualizes both the strength (*q*) and a property of the underlying function (*m*) of all associations of a given association type. Projecting all associations of a specific association type into the two-dimensional “association space” thus represents an individual fingerprint of the relationship between two ecosystem constituents which we refer to as an association profile (that is the distribution of individual strength and monotonicity estimates for all associations per association type). A visual inspection of the association profiles exhibited differences between the association types, especially among the biotic constituents of the ecosystem. We thus aimed to test for differences in association profiles with a focus on the biotic association types. To this end, we separately analyzed the differences of strength *q* and monotonicity *m* for each pair of association types by running a Bonferroni corrected pairwise Wilcoxon signed rank test. This step allows to assess whether certain constituents are stronger associated with others and whether there are differences in the degree of monotonicity of the associations. Ecologically speaking we tackled the question whether certain organismal groups are generally more dependent on each other (for instance: “are bacteria in general more dependent on plants than on fungi?”) and in which way organismal groups are associated to each other (for instance: “are bacteria in general more positively associated to plants than to fungi?”). We further adopted the framework of dynamic rangeboxes (*dynRB*) for evaluating the similarity between association profiles (Junker and others 2016). *dynRB* quantifies the overlap of two association profiles in the two-dimensional (that is the *q* and *m*) space by considering the full distribution of associations. We used the R-package *dynRB* v0.16 (Schreyer and others 2021) to estimate the portion of overlap *port(A,B)* between association profiles and converted the resulting portions into dissimilarities via 1 – *port(A,B)*. We then performed a hierarchical clustering on the resulting dissimilarity matrix to visualize and quantify the similarity between different association profiles. Support of the single clusters was assessed through multiscale bootstrapping across 10,000 bootstrap replicates using the R-package *pvclust* v2.2–0 (Suzuki and Shimodaira 2006). The analysis answers the question whether—on average—central properties of association types differ, that is, the strength and monotonicity (for instance: “differ plant–bacteria associations in their properties from bacteria–fungi associations?”).

**Ecosystem coupling** The coupling of an ecosystem is usually expressed as the mean strength of pairwise (symmetric and undirected) associations among ecosystem constituents and is then compared against a confidence interval derived from a randomly generated null model (Risch and others 2018; Ochoa-Hueso and others 2021). The initial concept of ecosystem coupling builds upon correlations (for example Spearman’s or Tjøstheim’s rank correlation coefficients) that are not capable of detecting non-monotonic associations. Using *qad* on our dataset allowed to incorporate non-monotonicity and direction of dependence to the concept of ecosystem coupling but necessitated slight adaptations in the calculation of ecosystem coupling as compared to the initial concept (Risch and others 2018). The level of detected coupling is highly scale dependent, both in space and time, and can greatly vary according to intrinsic characteristics, such as randomness/noise and the number of components being considered (Ochoa-Hueso and others 2021). As we used an untargeted approach that considers the associations among numerous biotic and abiotic constituents in a developing ecosystem, we calculated ecosystem coupling in two different ways. We first estimated ecosystem coupling as proposed by Ochoa-Hueso and others (2021) including all associations, irrespective of their significance. Second, we restricted the estimate of ecosystem coupling to only include significant associations to focus on the properties of the realized associations excluding random noise in the data. Then, we adopted the initial framework of coupling as follows: 1) We ran a null model with 999 permutations of our dataset separately for each association type and calculated the random coupling as the mean association strength *q* of all associations based on the null model. 2) We calculated the observed coupling for each association type individually as the mean association strength *q* based on the real dataset. We calculated the observed coupling once using all associations and once using significant associations only. 3) We then compared the proportion of observed significant associations to the proportion of significant associations that occur by chance and performed a G^2^-test to assess whether specific association types comprise more significant associations than expected by chance. A higher proportion of significant associations than expected by chance would be indicative of orderly processes within the ecosystem and thus coupling. 4) For each association type separately, we subtracted the estimate of random coupling (that is the mean *q* of all associations of the null model in the respective association type) from the *q*-value of each observed association (that is once for all associations, once for significant associations only). This step normalizes the coupling estimates around 0 where observed coupling equals randomly expected coupling. Respectively, normalized coupling estimates < 0 indicate weaker and values > 0 indicate stronger association than expected by chance. We then checked whether the distribution of coupling values deviates from 0 using a two-sided and a one-sided significance test representing significance levels of *alpha* < 0.05 and *alpha* < 0.025) We calculated an index of relative coupling, defined as the ratio of the mean observed association strength *q* (including only significant associations) divided by the mean strength *q* of all associations from the null model. This ratio is an easy to interpret measure that depicts how strongly coupled two (a-)biotic constituents are in comparison to random chance. It further allows comparisons of the relative coupling across all association types.

**Figure B2.1:**
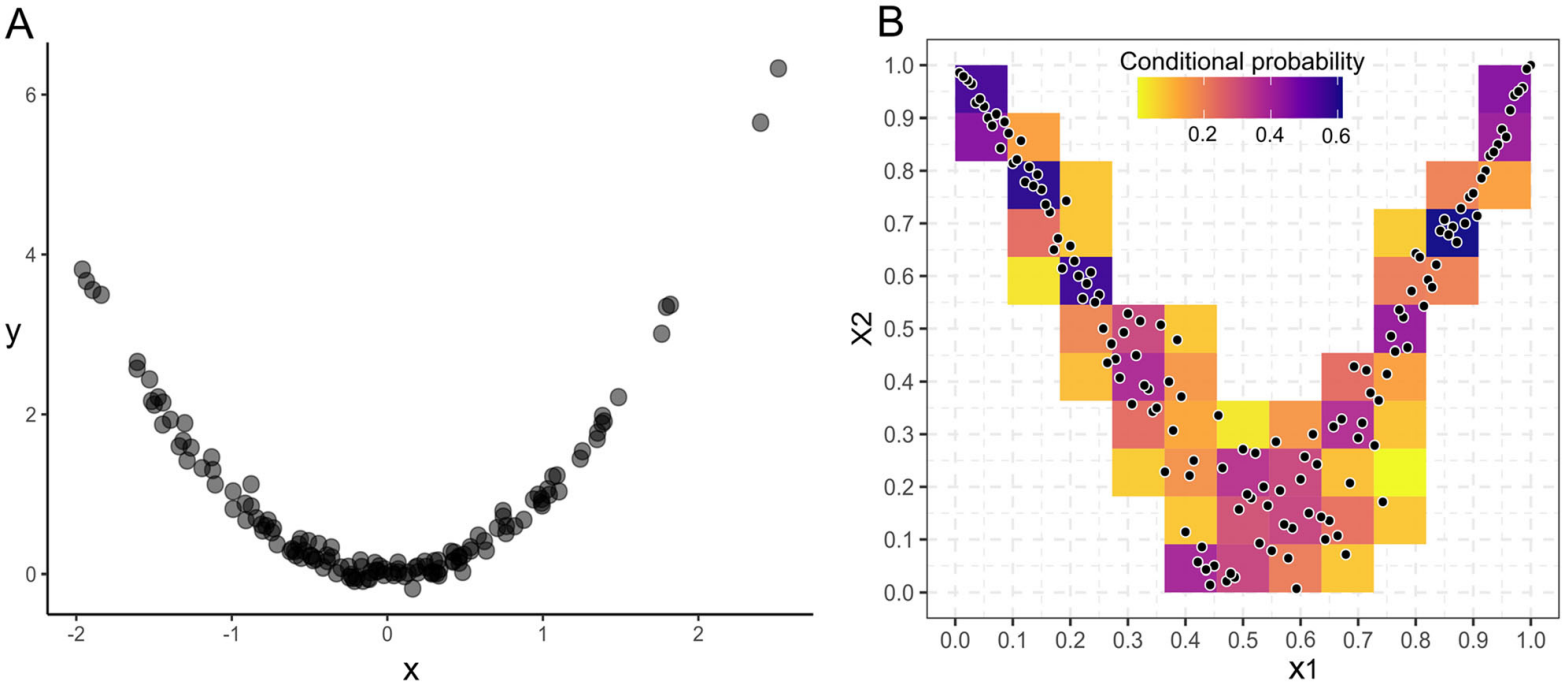
The non-monotonic association between X and Y visualized as a two-dimensional scatterplot** A** and as an empirical checkerboard copula** B**). To obtain the empirical checkerboard copula, observations **A** are transformed into normalized ranks **B**. Thus, the information on the marginal distribution of the observations is separated from the dependence structure, the latter is the copula. The empirical checkerboard copula is split into horizontal and vertical strips. The mass distribution of observations for each strip can be estimated and sums up to 1 in each strip (as indicated by the color gradient, darker colors indicate higher mass density, that is, higher conditional probability for an observation to fall within a certain interval of the strip).

**Figure B2.2:**
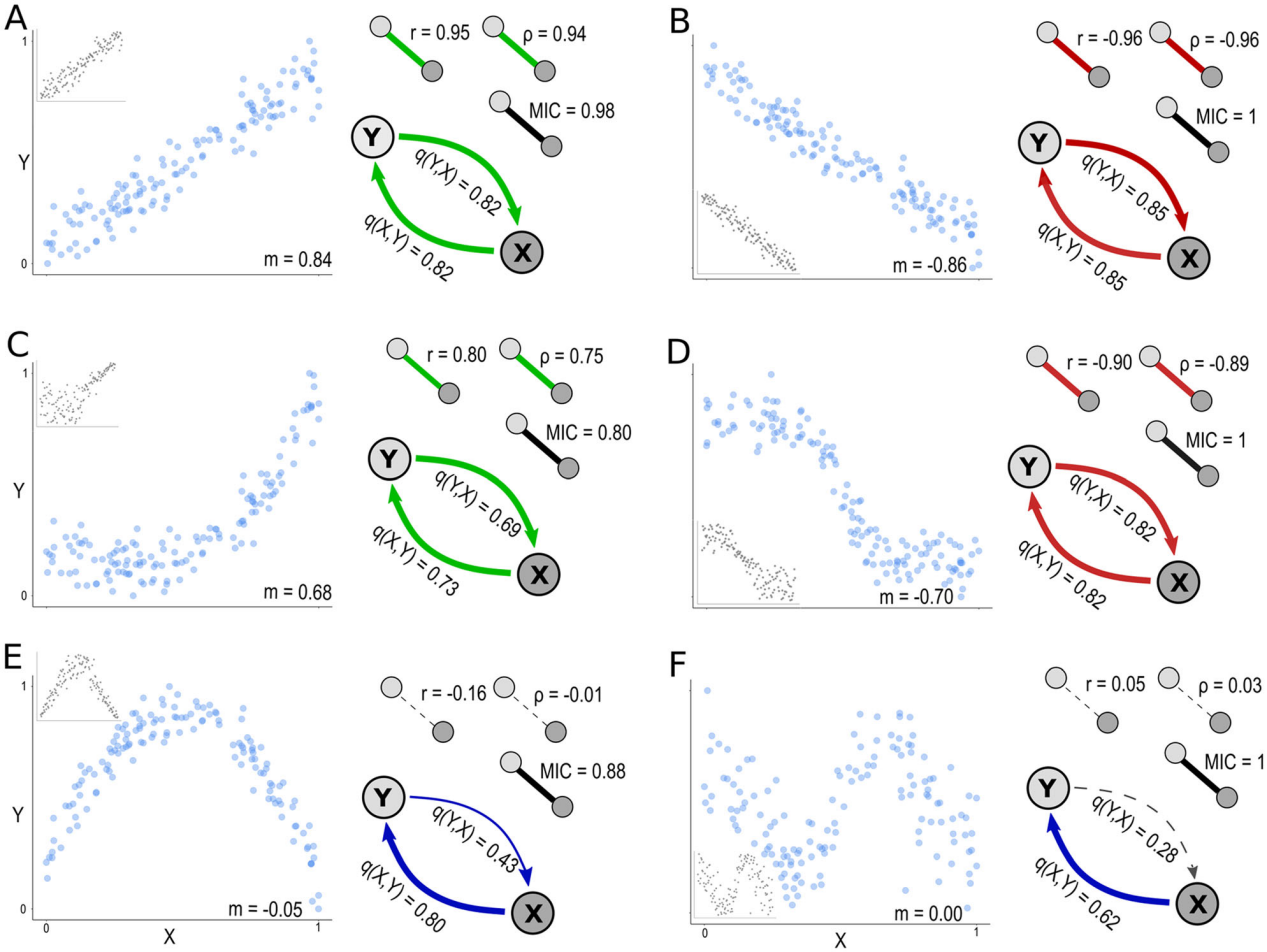
From associations to networks. Various monotonic and non-monotonic associations between random vectors X and Y visualized as two-dimensional scatterplots (blue circles) and as rank-based scatterplots (gray circles, in inset). Small networks are used to visualize the relationship between X and Y as summarized by different symmetric dependence measures (Pearson’s r, Spearman’s ρ, MIC), as well as the asymmetric dependence measures *q(X,Y)* and *q(Y,X)*. Lines represent undirected (that is symmetric) dependencies; arrows represent directed (that is asymmetric) dependencies. The coloration of the arrows indicates the direction of the association as estimated by the respective correlation coefficient; for *qad*, the monotonicity was estimated by the index of monotonicity *m*. Green color indicates positive monotonic, red negative monotonic and blue non-monotonic associations (for Pearson’s *r* the colors indicate linear and nonlinear associations, respectively). Black lines indicate that there is no estimate on the monotonicity of association possible (MIC), or indicate non-significance. Solid lines represent significant and dashed lines non-significant dependencies. While Person’s *r* and Spearman’s *ρ* can detect and quantify the dependence of linear (**A**,** B**) and Spearman’s *ρ* can detect and quantify nonlinear monotonic associations (**C**,** D**), both measures fail to identify non-monotonic associations (E, F). The maximum information criterion (*MIC*) detects all monotonic and non-monotonic associations but does not provide an estimate on asymmetry and direction of monotony as indicated by network edges. *qad* can accurately detect and quantify all monotonic, and non-monotonic associations among variables, provide an estimate on the (a) symmetry, and complemented by the index of monotonicity *m,* provide an estimate on the monotonicity of association.

Box 2: A Brief Intro to *qad-*Analysis The copula-based dependence measure *qad* detects and quantifies all scale-invariant dependence between pairs of random variables/features (Junker and others 2021a; Griessenberger and others 2022) and thus allows to quantify dependences regardless of the type of the regression function, that is, monotonic as well as non-monotonic associations can be detected. Furthermore, instead of quantifying the overall dependence between *X* and *Y* in a symmetric way, *qad* provides two measures of directed (asymmetric) dependence, *q(X,Y)* and *q(Y,X)*. The underlying method does not have any requirements on the underlying distribution of the data and is able to capture dependence in pairwise associations far beyond linear or monotonic relationships. For instance, in an association in the form of a parabola (Box 2; Figure B2.1A, B2.2E) or sinusoidal curve (Box 2; Figure B2.2F), the dependence structure is clearly non-monotonic and asymmetric; commonly used dependence measures, such as Pearson’s *r* or Spearman’s *p*, will not detect any dependence. Both afore-mentioned structures, however, have in common that variable Y is more dependent on X than *vice versa*, that is, knowing X provides more information on Y than *vice versa*. Contrary to symmetric measures providing only one coefficient of correlation summarizing the overall dependence between two variables, *qad* returns two dependence values accounting for potential asymmetry in dependence: $q\left(X,Y\right)$ denoting the dependence of Y on X (or, equivalently, the influence of X on Y), and $q\left(Y,X\right)$ denotes the dependence of X on Y. The two measures $q\left(X,Y\right)$, and $q\left(Y,X\right)$ can attain values between 0 and 1 (with 0 representing independence and 1 representing complete dependence, that is, knowing X means knowing Y without error) and are associated with *p*-values that are estimated via a permutation test. Further, the asymmetry of dependence is calculated as $a= q\left(X,Y\right)-q\left(Y,X\right)$. The following lines of code illustrate the use of *qad* on a simulated dataset.
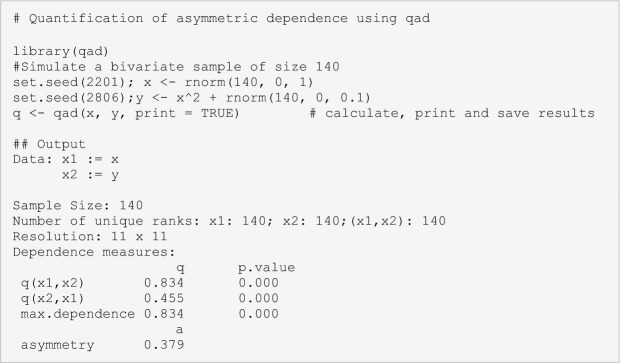

In contrast to other recent dependence measures capable of capturing the strength of both, monotonic and non-monotonic associations, between two variables (for example MIC (Reshef and others 2011), Figure B2.2E, F), *qad* additionally provides information on the direction and asymmetry of dependence. However, *qad* as implemented in the R-package qad (Griessenberger and others 2021) does not inform on the form of the relationship, that is, whether an association is positively or negatively monotonic, or globally non-monotonic. To evaluate the relative strength of monotonicity and the direction of the relationship between X and Y (whether the association is positively monotonic, non-monotonic, or negatively monotonic), we propose the index of monotonicity *m.* The index is based on the distance D1 (Trutschnig, 2011; available in the R-Package *qad*) between the estimated empirical copula (X,Y) and the co-monotonicity copula M (describing perfect positive association) as well as the counter-monotonicity copula W (perfect negative association). *m* attains values between -1 and 1 (with *m* = 1 indicating a strictly positive monotonic, *m* = -1 indicating a strictly negative monotonic, and a value of *m* ~ 0 indicating a non-monotonic dependency between X and Y, or none). All *qad*-based dependence estimates can be complemented with this measure to inform about the degree and direction of monotonicity of the association. Thus, *q* and *m* independently inform about two important properties of associations, strength and form. The following lines of code provide a function to quantify the index of monotonicity *m*.
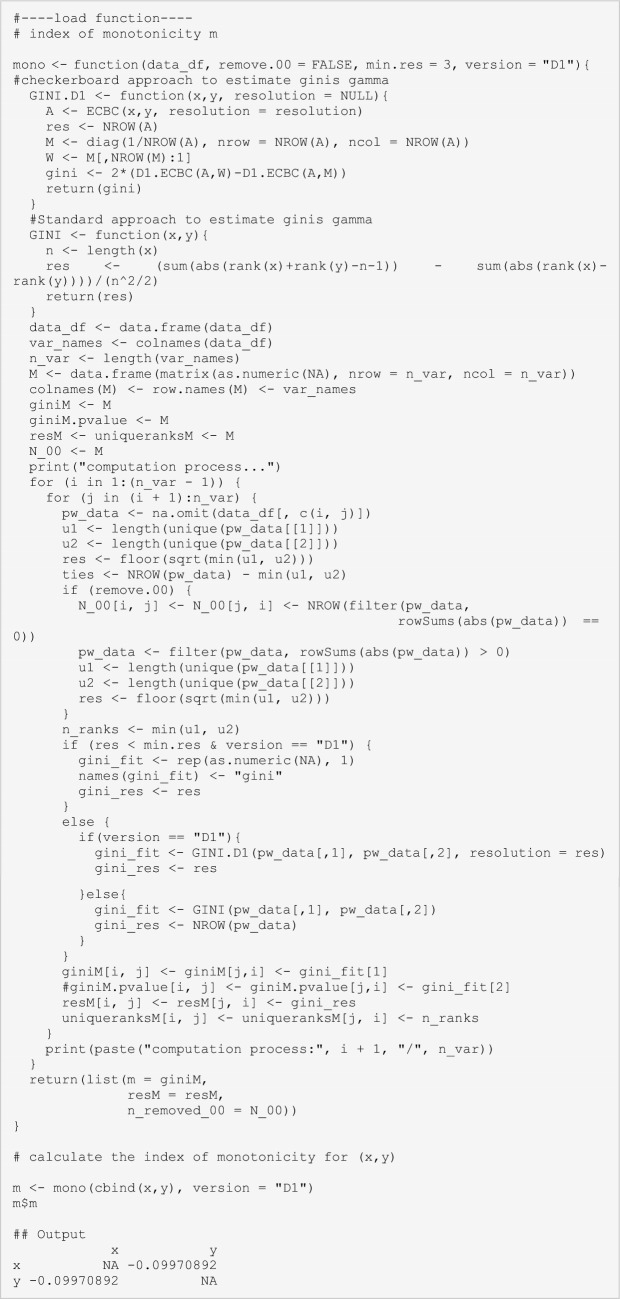

Given a dataset with more than two variables, *qad* can be applied to quantify all pairwise dependencies and allows an interpretation similar to that of an asymmetric correlation matrix. Based on this matrix, directed, asymmetric networks can be calculated:
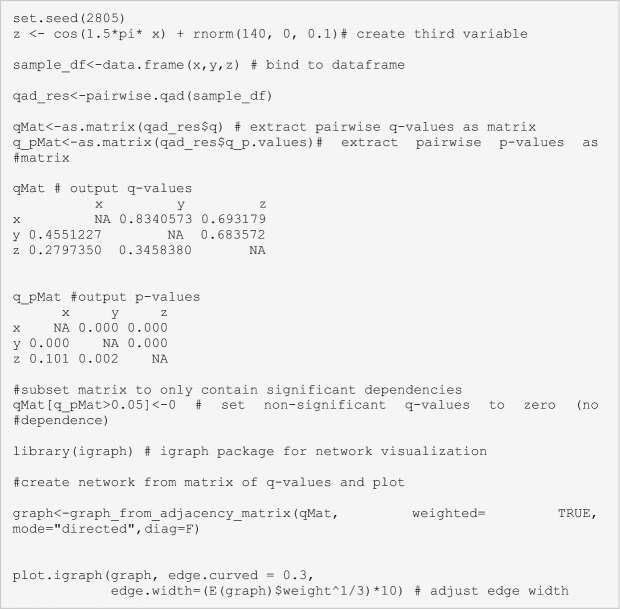

## Results

**Figure 1:**
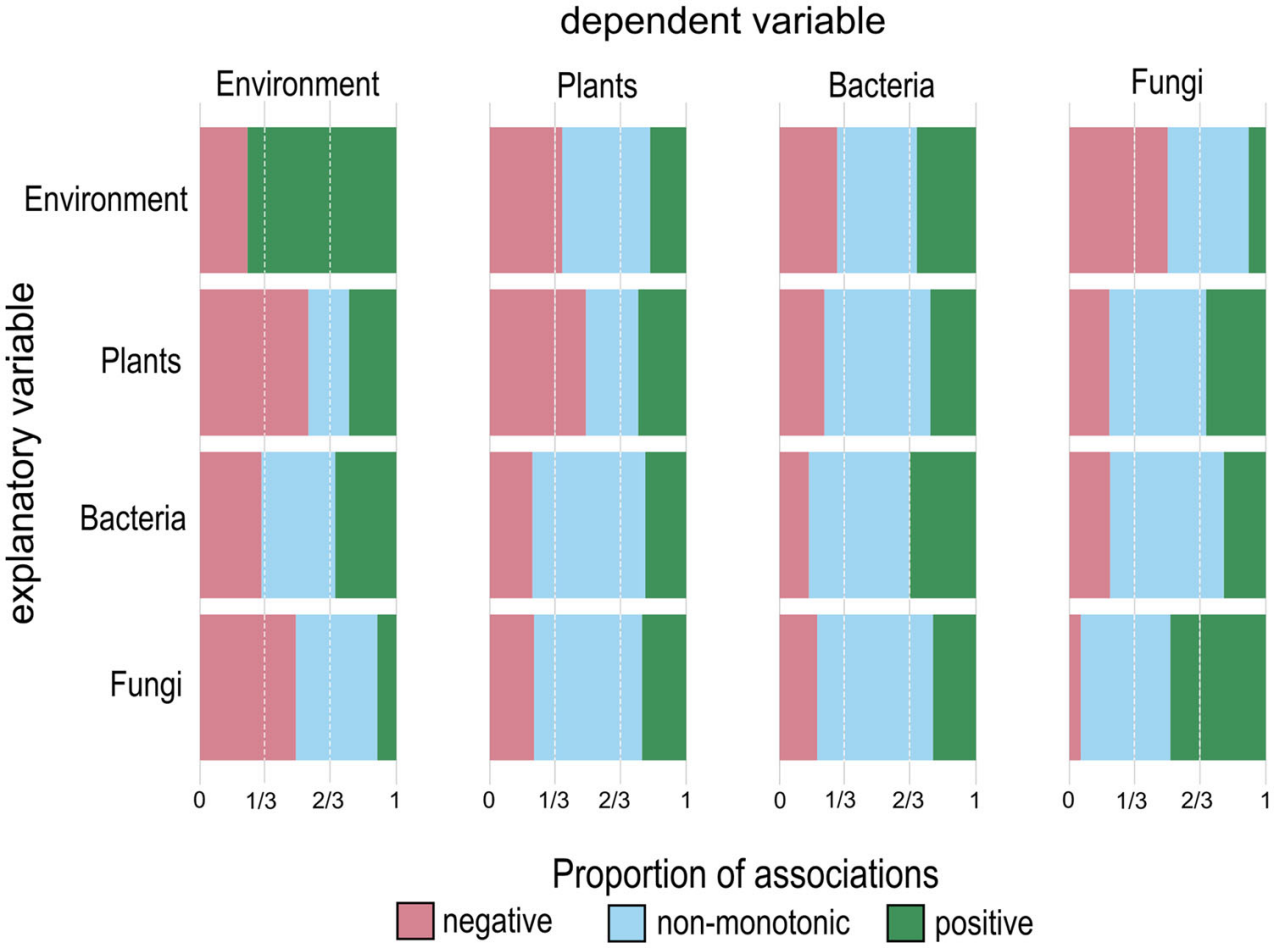
Proportion of positive (green), non-monotonic (blue), and negative (red) associations among organismal constituents and environmental variables along the successional gradient. In contrast to symmetric correlation-based measures, each *qad* calculation results in two directed values. For instance, the association between a fungal ASV and a plant species yields the two directed associations of Fungus → Plant and Plant → Fungus (that is explanatory → dependent). All directed dependences are assigned to a specific association type. For instance, the directed dependence between a plant and a fungus is assigned to the two association types Plants → Fungi (explanatory plant to dependent fungus) and Fungi → Plants (explanatory fungus to dependent plant).

**Community-wide association analysis** For the analysis of the proportion of non-monotonic associations along the whole gradient, we tested the strength and monotonicity of *n* = 447,758 pairwise associations, that is, *n* = 895,516 directed dependencies were calculated, of which *n* = 63,229 (7.06%) were statistically significant. The total number of significant dependencies was not evenly distributed among association types. Bacteria were the organismal group with the highest taxonomic richness, and as such, most significant associations occurred between two bacterial partners (*n* = 43,738), whereas the fewest significant associations were detected in the association types Environment → Environment and Plant → Environment with total of *n* = 29 significant associations each (for a complete table see Supplement 1). In total, most siginificant associations *n* = 32,404 (51.2%) were classified as non-monotonic associations, whereas *n* = 20,676 (32.7%) were classified as positive and *n* = 10,149 (16.1%) as negative associations. The proportion of positive, negative, and non-monotonic associations significantly varied among the association types (G-test of independence, *p* < 0.01). The proportion of positive monotonic associations per association type ranged from 8.8% (Environment → Fungi) to 75.9% (Environment → Environment), while the number of negative monotonic associations ranged from 5.7% (Fungi → Fungi) to 55.2% (Plant → Environment) and the number of non-monotonic associations ranged from 0% (Environment → Environment) to 59.0% (Fungi → Bacteria, Figure 1). By separating the gradient into early and late successional stages, we found a significantly higher proportion of non-monotonic associations (paired Wilcoxon sign-ranked test_*m*_: *p* = 0.025, Supplement 1) and a lower proportion of positive associations (*p* = 0.003) within the late successional stages as compared to early successional stages.

**Figure 2:**
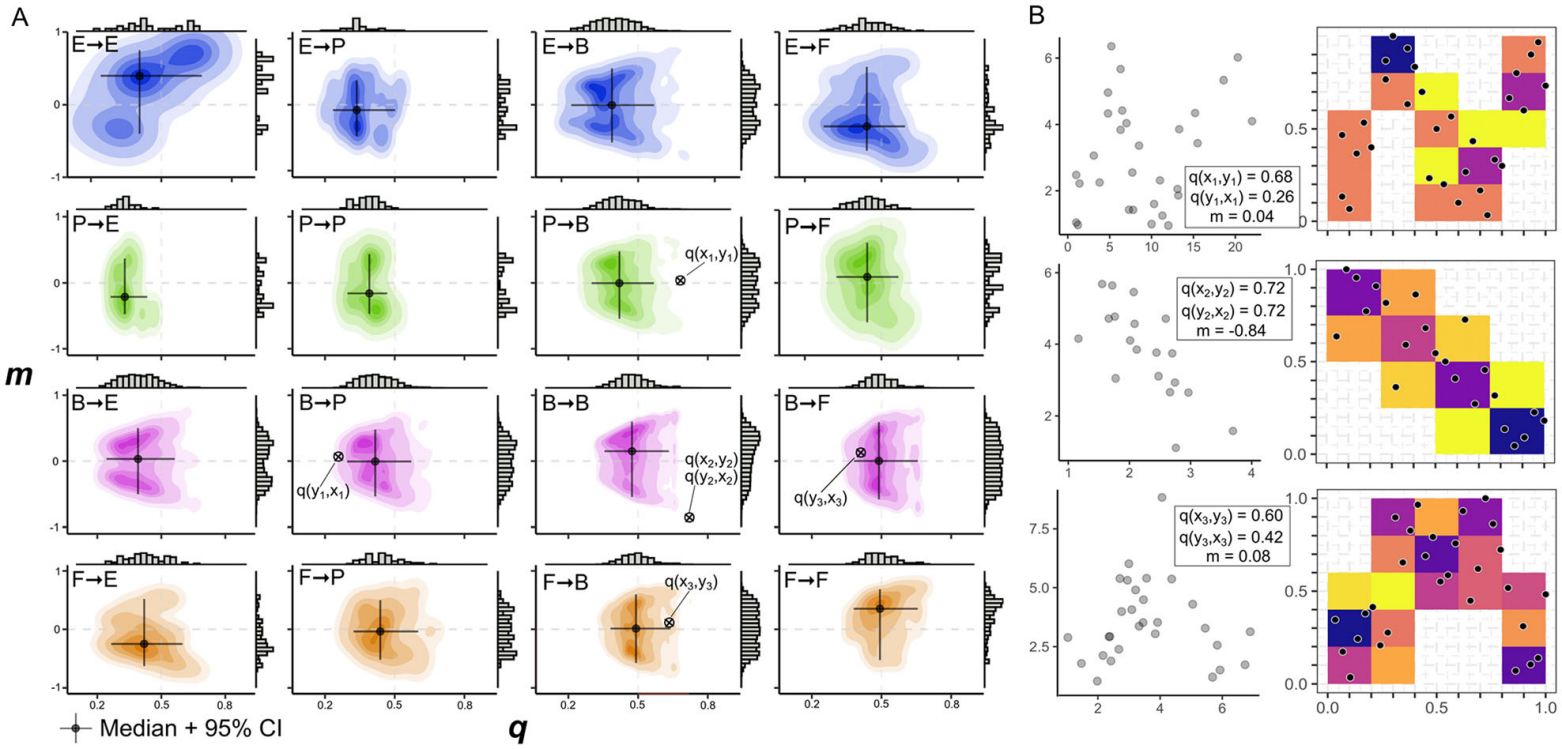
Association space:** A** Directed dependence (*q*) and the corresponding index of monotonicity (*m*) of the association types projected in a two-dimensional association space. Shaded areas show density distribution of all pairwise associations in the association space defined by *q* and *m.* Darker colors indicate a higher density of associations in the two-dimensional space. Black crosses indicate the median *q* and *m* for each association type with 95% confidence intervals. Highlighted directed dependences represent selected associations from B. Letters indicate the first letter of the ecosystem constituents (that is Bacteria: B; Environment: E; Fungi: F; Plants: P; Explanatory variable → dependent variable).** B** Examples of real-world monotonic and non-monotonic associations in our dataset shown as scatter plots (left) and as empirical checkerboard copulas (right, darker colored squares indicate a higher point density). The directed dependences *q*(X, Y) and *q*(Y, X) of each association are projected onto the two-dimensional space of the respective association type in A). Top: A non-monotonic, sinusoid association between the plant species *Scorzoneroides helvetica* on the x-Axis and a Betaproteobacterium on the y-axis. Center: A monotonic, negative association between a bacterium of the Comamonadaceae family on the x-Axis and a bacterium of the Caulobacteraceae family on the y-axis. Bottom: A non-monotonic, quadratic association between a taxonomically not resolved fungus on the x-Axis and a bacterium of the Acidobacteriaceae family on the y-axis.

**Figure 3:**
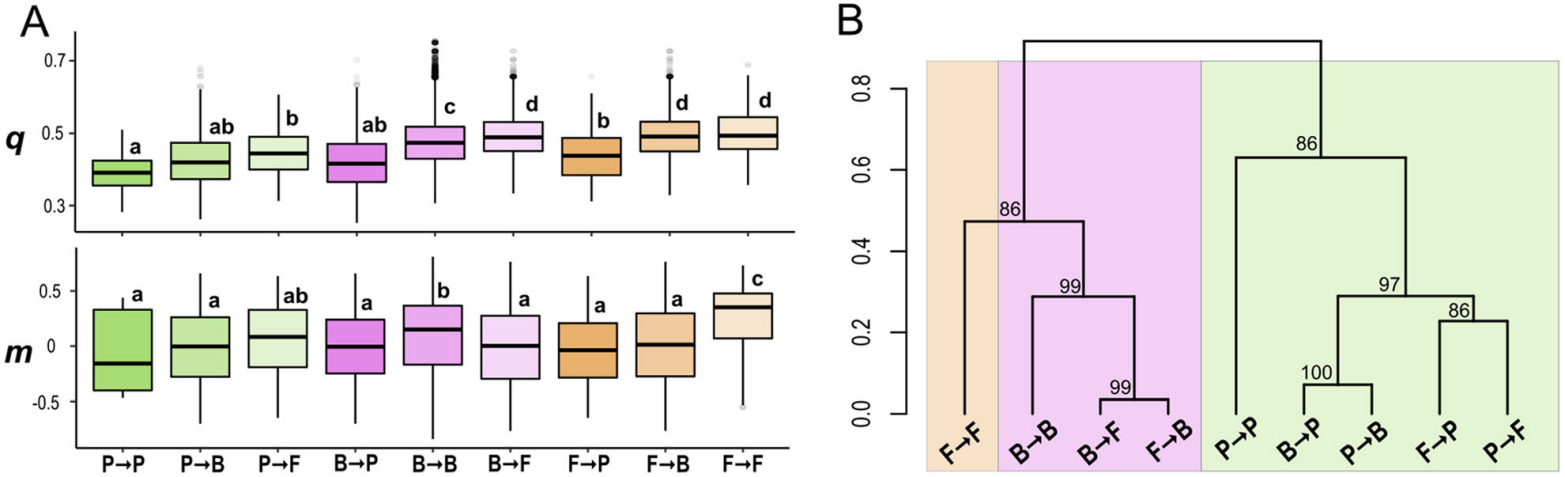
Association profiles of different association types (Bacteria: B; Environment: E; Fungi: F; Plants: P). ** A** Strength *q* and index of monotonicity *m* for each directed dependence estimate grouped by association type. Letters above boxplot indicate significant differences in mean values, as estimated by analysis of variance and subsequent post hoc test. ** B** Hierarchical clustering of biotic association types as calculated by the proportion of overlap between the associations in a two-dimensional space of joint distribution of *q* and *m.* Numbers at nodes depict probabilities supporting the topology of the dendrogram. Coloration indicates the presence of a specific organismal group within each cluster (that is subclusters including plant partners are colorized in green, fungal subclusters in orange, and bacterial subclusters in magenta).

**Specific association profiles among association types** The association types showed significant differences in strength (Wilcoxon pairwise sign-ranked test_*q*_: *p* < 0.01; Figure 2A; Figure 3A) and monotonicity (*p* < 0.01, Figure 2A; Figure 3A). The pairwise comparison among all association types revealed that Fungi → Fungi, Fungi → Bacteria and Bacteria → Fungi associations show on average the strongest dependence, whereas Plant → Plant, Plant → Bacteria and Bacteria → Plant associations harbored on average the weakest dependencies. Fungi → Fungi associations showed on average the highest values of monotonicity *m*. Different association types were characterized by individual association profiles in the two-dimensional *q-m* “association space” (Figure 2). The hierarchical cluster analysis of the association profiles emphasized significant differences among association types, too. Association types were separated into two distinct clusters based on whether the association type included a plant partner or not. Within their respective cluster, Fungi → Fungi and Plant → Plant associations were well separated from the other association types and Bacteria → Bacteria associations were substantially different from Bacteria → Fungi and Fungi → Bacteria associations (Figure 3B).

**Figure 4:**
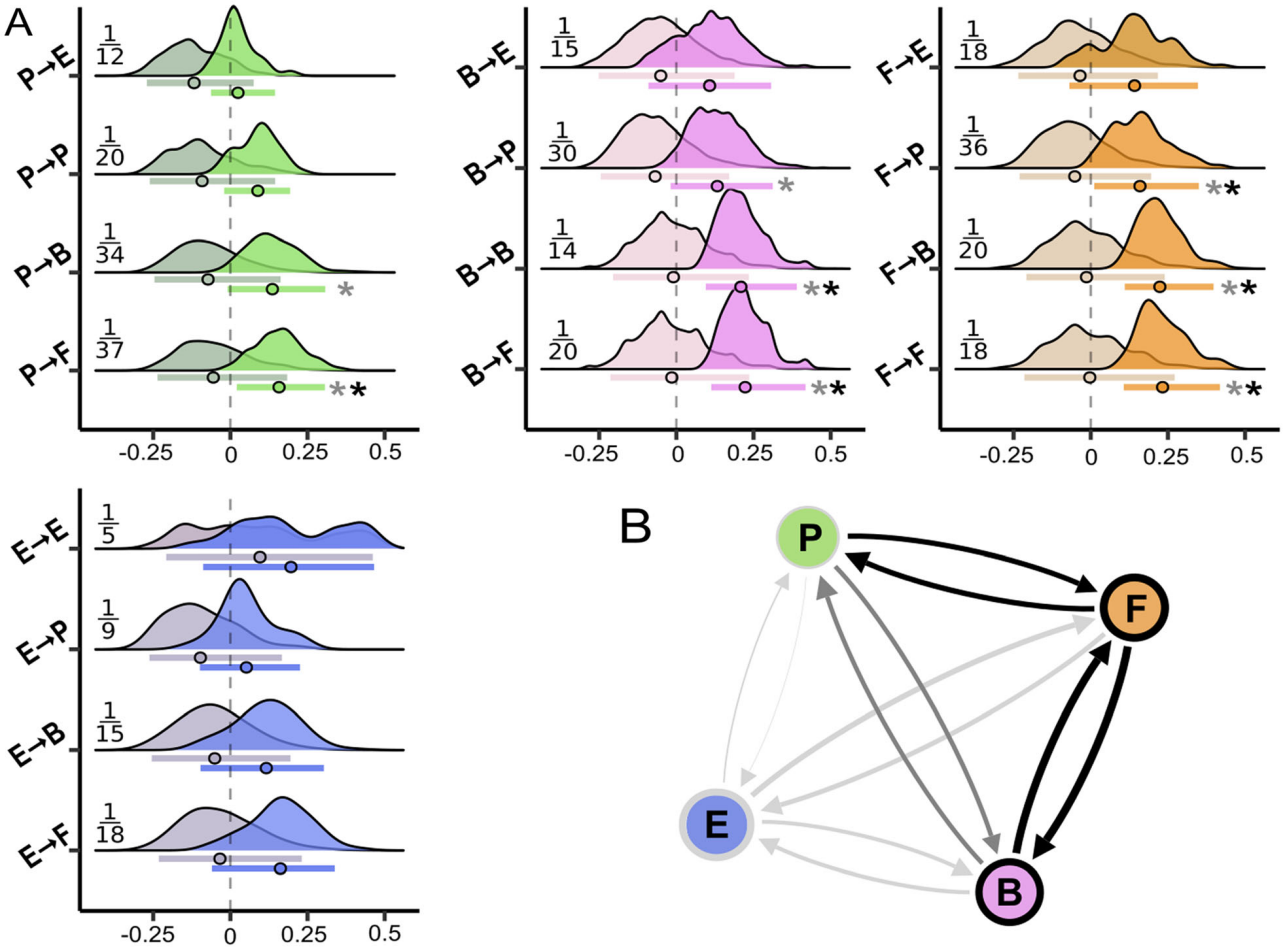
Directed coupling between (a-)biotic ecosystem constituents (Bacteria: B; Environment: E; Fungi: F; Plants: P). **A** Directed coupling per association type. Colored densities represent the normalized coupling of each association type. To assess normalized coupling, we subtracted the mean *q* of all associations of the null model in the respective association type from the *q*-value of each observed association. This step normalized the coupling estimates around 0 where observed coupling equals randomly expected coupling. Respectively, normalized coupling estimates < 0 indicate weaker and values > 0 indicate stronger association than expected by chance. Circles indicate the mean observed coupling and error bars represent the 95% interval of the normalized association strength. Asterisks mark whether the mean random coupling significantly differs from the observed coupling based on two significance levels: single gray asterisks indicate a one-tailed significance test with *alpha* < 0.05; additional black asterisks indicate that significance was additionally assessed based on a two-tailed test with *alpha* < 0.025. Saturated colors indicate the coupling of significant associations only, grayish colors of the same tone represent coupling based on all associations. Fractural numbers indicate the proportion of significant associations among all associations. **B** The relative (that is mean observed coupling of significant associations divided by mean of random coupling) directed ecosystem coupling between the (a-)biotic groups represented as a directed network. The thickness of the arrows is proportional to the directed coupling strength, the thickness of the border of single nodes is scaled to represent the coupling strength within the respective group. Black color indicates that the random coupling of the specific association type significantly differed at *p* < 0.025; gray color indicates *p* < 0.05 and light gray indicates no significant deviation of random coupling from observed coupling.

**Ecosystem coupling** Overall, we found a higher proportion of significant associations in our dataset than expected by chance (*X*^*2*^ = 6.28, *p* < 0.01) indicating a distinct coupling in the observed ecosystem. Specifically, we found more significant associations than expected by chance within each association type (Supplementary Table 2), which points at orderly connected abiotic and biotic features in the ecosystem. If all associations are considered (that is significant and non-significant associations), the coupling as expected by chance does not differ from the observed coupling in any association type, and only in Environment → Environment associations the observed coupling exceeds the random coupling, which is indicative of an ecosystem that is decoupled in large parts. However, when only significant associations are considered, the strength of random coupling was significantly lower than the observed coupling in six association types when performing a two-tailed test and in eight association types when a one-tailed test is performed (see Figure 4). The relative strength of directed coupling of the significant associations varied between different association types and ranged between 1.06 and 1.59. Environment → Environment associations (that is abiotic coupling) yielded a comparably high relative coupling of 1.53, while Fungus → Fungus and Fungus → Bacteria associations showed the highest biotic coupling of 1.59 (Figure 4, Supplementary Table 3). In general, any directed coupling that included plants as partner yielded comparatively low values of directed coupling, while we detected the lowest relative coupling (1.06) of all association types in Plant → Environment associations (Figure 4B).

## Discussion

Our results clearly demonstrate that non-monotonic associations are frequent (if not prevailing) in natural ecosystems and that different association types are characterized by specific profiles capturing their strength and monotonicity. In general, associations that include a plant partner differed from purely microbial associations and the profiles of intra-kingdom associations were well separated from inter-kingdom association types. These differences in association profiles are further reflected in varying degrees of directed coupling between the constituents of the ecosystem. We here extended the concept of ecosystem coupling with the inclusion of non-monotonic associations and directed dependence and are thus able to reveal a strong asymmetry in the relative coupling between plants and the environment. Therefore, our study emphasizes the relevance of so far understudied ecological non-monotonicity and serves as a call to action for ecologists to make use of new statistical tools that will take their analyses beyond the focus on symmetry and monotonicity.

**Ecological non-monotonicity –** *qad* in combination with the index of monotonicity *m* turned out to be a valuable tool for a complete characterization of associations regardless an underlying function type. Thus, *qad* unites the advantages of previous undirected approaches (for example machine-learning) to measure the strength of non-monotonic associations and extends their capabilities for the element of direction of dependence. For instance, machine-learning approaches have been used for predictions using complex ecological data, such as the links between soil bacterial community composition and environmental factors (Hermans and others 2020) and in trait-based predictions of species interactions for analyzing interaction networks (Pichler and others 2020). Fontaine and others (2021) have used machine-learning algorithms in combination with the maximal information coefficient (MIC, Reshef and others (2011)) and the maximum asymmetry score (MAS) for detecting non-monotonic associations between biotic and abiotic parameters. Congruent to the *qad*-based results of our study, they found a high proportion of non-monotonic associations between bacterial ASV and environmental variables. However, the combination of the *qad*-based dependence estimates *q* and monotonicity estimates *m* allows a more comprehensive and intuitive analysis of ecosystem-wide association than previous workflows, as it can also capture the direction of dependence. Thus, in contrast to standard correlation or machine-learning methods, *qad* quantifies the strength (*q*), the asymmetry (*a*), and important information on the function type (that is degree of monotonicity *m*) of an association independent from each other.

In our study, we used species abundances to discover statistically significant associations between pairs of co-occurring species and environmental parameters. While co-occurrence is clearly not evidence of ecological interaction, abundance data are informative of the performance of taxa (Ross and others 2023) and do contain deeper information on the determinants of a local species’ distribution than mere presence–absence data (Blanchet and others 2020). Thus, abundance-based association analyses that consider the full range of natural outcomes of relationships (that is monotonic and non-monotonic associations) between species or organismal group can serve as a powerful tool for ecologists to detect association patterns and to generate hypotheses on biologically relevant associations or global structuring mechanisms of specific association types. Accordingly, such community-wide analyses may contain more information about ecological communities and taxa than studies focusing on a single pair of interaction partners at a given place and time (Bronstein 1994). In our study, we found that different association types exhibit variable amounts of monotonic and non-monotonic associations. In Environment → Environment associations, for instance, we did not detect any non-monotonic associations, while association types that include a microbial partner generally show a higher proportion of non-monotonicity. Fungi → Fungi associations, for instance, show hardly any negative associations but are characterized by a high number of positive and non-monotonic associations. While monotonic associations between taxa can be explained by traditional species interaction models (that is mutualism, competition, etc.) or strong similarities in the species’ environmental requirements (Blanchet and others 2020), the reasons behind non-monotonic associations may be more complex and not follow classical interaction outcomes, especially under changing environmental conditions. Fontaine and others (2021) detected a high number of non-monotonic associations of bacterial ASV along an environmental gradient and attributed the prevalence of non-monotonicity to shifts in interactions between individual ASV, as well as opposing positive and negative environmental effects on co-occurrence patterns of bacteria. Our study system also represents a strong environmental gradient (Junker and others 2020, 2021b; Hanusch and others 2022) and thus gradual changes in the abiotic and biotic environment along the gradient may invoke non-monotonic relationships between organisms. For instance, it is well recognized that environmental harshness leads to facilitative interactions between species, while negative interactions prevail under benign conditions (Tielbörger and Kadmon 2000). Such environmentally driven changes in the interaction outcome have been shown to occur in a wide range of organismal groups ranging from plants to bacteria and fungi and can result in various non-monotonic associations between two partners (Kjær and others 2018; Velez and others 2018; Hammarlund and Harcombe 2019; Piccardi and others 2019). Although *qad* detects non-monotonic associations*,* we can only hypothesize about the underlying mechanisms that lead to specific non-monotonic associations. Thus, a careful examination of case-specific non-monotonicity through detailed investigation of individual associations is mandatory. We thus appeal for more efforts to study the underlying causes of ecological non-monotonicity and its role in maintaining the biodiversity of ecosystems in future case studies and experiments.

So far, most studies investigating the implications of non-monotonic associations have been focused on so-called dome-shaped relationships that represent a transition from a positive to a negative associations or *vice versa* (Yan and Zhang 2018b, 2018a). However, using *qad,* we can detect all kinds of associations without limitation to specific function types such as linear, exponential, or periodic. Although the index of monotonicity *m* informs about the direction and monotonicity of an association, it is impossible to make assumptions on the explicit function type of a specific non-monotonic association without taking a closer look at the individual relationship. We found that most significant non-monotonic associations involve at least one microbial partner, which confirms empirical and simulated results of previous studies (Weiss and others 2016; Hirano and Takemoto 2019). However, various causes for non-monotonic associations have been suggested preventing the generalization of potential explanations.

**Association profiles** The associations between individual organismal groups harbored distinct association profiles (that is average positions within the “association space”) that markedly differ from other association types. The two-dimensional profiles of purely microbial associations show little overlap with associations that involve a plant as a partner and inter-kingdom associations are well separated from intra-kingdom associations of the same organismal groups. Close associations between two organismal groups are usually interpreted to be likely driven by trophic interactions, with stronger associations occurring between taxa with more specialized interactions (Manning and others 2015). A previous study in the same glacier forefield found strong biotic dependences between soil inhabiting bacteria and fungi, especially in the older parts of the successional gradient where closed nutritional cycles have formed (Hanusch and others 2022). Most biogeochemical cycling processes depend on complex cascades of microbial interaction that comprise highly specialized mechanisms such as antibiosis, metabolite exchange, or signaling chemotaxis (Nazir and others 2010; Velez and others 2018), which may explain the strong associations between microbial partners in our results. On the other hand, associations between microbial and plant partners were characterized by comparatively weak associations. Previous studies have found only limited direct interactions between plants and the majority of the belowground microbiome, and soil microbiota tend to associate opportunistically with a wide range of plant taxa (Fierer 2017). Thus, our study confirms previous analyses that revealed only weak or no dependence between plant species and microbial abundances of the soil microbiome (Nunan and others 2005; Singh and others 2007; Lekberg and Waller 2016; Tedersoo and others 2016).

**Ecosystem coupling** If all associations are considered (that is significant and non-significant associations), all association types between the ecosystem constituents are decoupled. Ochoa-Hueso and others (2021) interpret the decoupling of constituents as an entropic configuration in response to disturbances, high (a-)biotic stress levels or other stochastic events and advocate to consider the developmental history of an ecosystem when interpreting estimates of ecosystem coupling. Primary successional gradients, especially within alpine glacier forelands, are characterized by high levels of abiotic stress, a high frequency of disturbance events, and rapid environmental and biotic changes (Ficetola and others 2021). We thus assume that the detected decoupling is presumably a result of the differential responses of species to rapid changes in the environment and stochastic events that occur over succession. However, the authors also mention that especially the level of coupling among biotic constituents can be influenced by changes in mutualistic and antagonistic behavior of species and is in general highly dependent on the scale at which it is investigated. While our *qad*-based approach can incorporate stable (that is monotonic) and a wide range of changing (that is non-monotonic) associations into the calculation of ecosystem coupling, the vast number of considered associations in our untargeted approach may have also contributed to a decrease in the level of detected coupling. Furthermore, the spatial scale of our study covers the whole successional gradient of our study site, which may lead to decoupling as our approach integrated over a number of distinct successional stages with a specific set of interacting species. When splitting the data into early and late successional stages, we found higher proportions of non-monotonic and positive associations in later successional stages, which indicates that non-monotonicity and ecosystem coupling responds to ecosystem complexity. Thus, future investigations should study coupling at smaller spatial scales within a successional gradient.

In a second step, we only considered significant associations for the calculation of ecosystem coupling focusing on ecologically relevant associations. Here, soil inhabiting bacteria and fungi show the highest coupling in our results, while associations that include a plant partner show a comparatively weak coupling. The directed coupling between plants and the environment, although generally weak, revealed a marked asymmetry in coupling strength, indicating that plants are stronger coupled to environmental features than vice versa. The asymmetric coupling probably is a product of the highly specific environmental conditions that certain plant species require over succession (Ficetola and others 2021; Junker and others 2021b). Environmental features often follow monotonic functions during primary succession, such as a gradual accumulation of soil nutrients or a decrease of soil pH through acidification with successional age (Junker and others 2020; Hanusch and others 2022). The abundance of plant species, however, will change over the course of succession due to the ongoing alteration of the abiotic environment (Walker and others 2010). For instance, late successional species establish only after abiotic parameters allow a successful establishment. In contrast, pioneering species that reach their abundance optima during early stages of succession will gradually be replaced by species that are better adopted to environmental conditions that occur in later successional stages (Chang and HilleRisLambers 2016; Hanusch and others 2022) resulting in non-monotonic functions between pioneering plant abundances and environmental variables. These non-monotonic associations ultimately lead to an asymmetric coupling in which plant species are more dependent on the environment than environmental variables depend on specific plant species. This result exemplifies the gain in analytical power and great potential for the analysis of ecological datasets that comes along with the inclusion of non-monotonicity, especially when dealing with spatial or temporal data.

## Conclusion

Our results suggest that *qad* generates unexplored and robust insights into the complex relationships that structure natural ecosystems. Based on its universality and straightforward applicability, we argue that the estimation of *qad*-based directed dependence will help ecologists to identify novel associations in their datasets and that our approach can generate meaningful insights within ecology and related scientific fields. Our study provides a broad overview on the frequency and importance of non-monotonic associations in natural ecosystems. It is, however, crucial to validate and verify the identified associations to determine whether they are the result of interactions between pairs of taxa or reflect responses to environmental parameters leading to non-monotonic associations, too. Our data paired with a critical evaluation of the identified associations will reform our monotonicity-centric perception and will allow new insights into ecosystem functioning, into species co-existence, and into the causal mechanisms and the importance of ecological non-monotonicity.

### Electronic supplementary material

Below is the link to the electronic supplementary material.

Supplementary file1 (XLSX 19 KB)

Supplementary file2 (DOCX 13 KB)

## Data Availability

Raw sequences of next-generation 16S rRNA gene amplicon sequencing are available at the NCBI Sequence Read Archive (SRA) under the BioProject accession PRJNA701884 and PRJNA701890. Raw floristic and environmental data are available as a Mendeley Data repository under https://doi.org/10.17632/xkv89tbftc.2.
