# Supplementary material for: Exploring the Frequency and Distribution of Ecological Non-monotonicity in Associations among Ecosystem Constituents
Source: Ecosystems. 2023 Aug 14;26(8):1819–40. doi: 10.1007/s10021-023-00867-9 (PMC10721710; doi:10.1007/s10021-023-00867-9)
Supplement: Supplementary file 2 — Supplementary file2 (DOCX 13 KB) [file 10021_2023_867_MOESM2_ESM.docx]

ECOSYSTEMS MANUSCRIPT INFORMATION SHEET

MANUSCRIPT NUMBER: ECO-23-0008.R1

TITLE: Exploring the frequency and distribution of ecological non-monotonicity in associations among ecosystem constituents

AUTHORS: Hanusch, Maximilian; He, Xie; Janssen, Stefan; Selke, Julian; Trutschnig, Wolfgang; Junker, Robert

CORRESPONDING AUTHOR:

Prof. Robert Junker

Department of Biology

University of Marburg

Marburg 35037

Germany

FAX:

PHONE:

EMAIL: [robert.junker@uni-marburg.de](mailto:robert.junker@uni-marburg.de)

RECEIVED 12-Jan-2023; ACCEPTED 06-Jul-2023

COLOR FIGURES: 7

COMMENTS:
